# The effect of increased mobility on SARS-CoV-2 transmission: a descriptive study of the trends of COVID-19 in Zimbabwe between December 2020 and January 2021

**DOI:** 10.11604/pamj.2021.39.125.28794

**Published:** 2021-06-15

**Authors:** Grant Murewanhema, Trouble Victor Burukai, Lameck Chiwaka, Fabian Maunganidze, Davison Munodawafa, William Pote, Jacob Mufunda

**Affiliations:** 1 Unit of Obstetrics and Gynaecology, Department of Primary Health Care Sciences, Faculty of Medicine and Health Sciences, University of Zimbabwe, PO Box A178, Avondale, Harare, Zimbabwe,; 2 Department of Anatomy, Faculty of Medicine and Health Sciences, Midlands State University, Gweru, Zimbabwe,; 3 Department of Surgery, Faculty of Medicine and Health Sciences, University of Zimbabwe, Harare, Zimbabwe,; 4 Department of Physiology, Faculty of Medicine and Health Sciences, Midlands State University, Gweru, Zimbabwe,; 5 Ethnobiology-based Drug Discovery, Research and Development Trust, Gweru, Zimbabwe,; 6 Department of Community Medicine, Faculty of Medicine and Health Sciences, Midlands State University, Gweru, Zimbabwe,; 7 Department of Physiology, Faculty of Medicine and Health Sciences, Great Zimbabwe University, Masvingo, Zimbabwe

**Keywords:** SARS-CoV-2, COVID-19, trends, Zimbabwe, second wave, mutants, pandemic

## Abstract

**Introduction:**

when the first cases of COVID-19 were reported in Zimbabwe in March 2020, the local outbreak was characterised by an insidious increase in national caseload. This first wave was mainly attributable to imported cases, peaking around July 2020. By October 2020, the number of cases reported daily had declined to less than 100 cases per day signalling the end of the first wave. This pattern mirrored the global trends. In December 2020, reports of new COVID-19 variants emerged and coincided with the beginning of the second wave within the ongoing pandemic. This paper reports on the analysis conducted on the new wave of COVID-19 beginning December 2020 to January 2021. The objective of this study was to document the evolving presumptive second wave of the COVID-19 pandemic in Zimbabwe from December 2020 to January 2021.

**Methods:**

this is a retrospective analysis of secondary data extracted from the daily situation reports published by the Ministry of Health and Child Welfare, Zimbabwe and World Health Organization Country Office, Zimbabwe. The period under consideration started from 1^st^ December 2020 to 31^st^ January 2021.

**Results:**

there was a 333% increase in the number of confirmed COVID-19 cases starting 1^st^ December 2020, to 31^st^ January 2021. These new cases were mainly attributed to community transmission though there were a few imported cases. There was a 439% increase in the absolute number of deaths; however, the case fatality rate remained low at 3.6%, and comparable to that from other countries. Harare, Bulawayo and Manical and provinces accounted for 60% of the case burden, with the other seven provinces only accounting for 40%. By mid-January, the number of incident COVID-19 cases started to decline significantly, to levels similar to the residual levels seen during the first wave.

**Conclusion:**

the second wave, which lasted a period of less than 2 months, had a steep rise and sharp decline in the incident cases and fatalities. The steep rise was attributable to increased mobility, with a consequent increase in the chains of community transmission. The declines, noted from mid-January 2021, may be partly attributable to a strict national lockdown, though more in-depth exploration of the drivers of transmission is needed to tailor effective interventions for future control. Differentiated strategies maybe needed according to the case burdens in the different provinces. In anticipation of further waves, the introduction of safe and effective vaccines might be the game changer if the vaccines are widely availed to the population to levels adequate to achieve herd immunity. Meanwhile, infection prevention and control guidelines must continue to be observed.

## Introduction

Zimbabwe recorded its first case of COVID-19 in March 2020 [1]. The outbreak in Zimbabwe took a very insidious course at the onset, unlike other countries where exponential case burden increases occurred from the outset [2]. Mathematical projections postulated that Zimbabwe and other African countries would suffer the greatest from the COVID-19 pandemic [3]. A retrospective analysis of data from the Ministry of Health and Child Care (MOHCC) showed that four months after the onset of the pandemic, Zimbabwe had not reached 1 000 confirmed cases [4]. The majority of the cases in the first wave were diagnosed among those who had visited other countries and/or returning residents [4]. The daily situation reports released by the MOHCC show that by the end of August 2020, the numbers of new cases had significantly dropped.

The government of Zimbabwe responded to the first wave by introducing a very strict lockdown, banning all non-essential movements, closing borders, schools and other amenities, with strict traffic check-points [1]. This response was well coordinated with other Southern African countries also instituting similar measures. World Health Organisation (WHO) guidelines for infection prevention and control were adopted and implemented in Zimbabwe. As the number of new and active cases started to decline, the restrictions, especially the lockdown, were gradually relaxed over a period of five months. With time passing, the population became complacent and people began to move about freely [5]. Compliance with WHO guidelines was compromised. The borders between South Africa and Zimbabwe were partially opened for trade and essential human travel especially during the month of December, with a corresponding increase in travel between the two countries. The partial opening of air travel, also saw an increase in the number of visitors from the United Kingdom, Europe and other countries.

A spike in the number of cases was noted in December 2020, reaching its peak in January 2021, as noted from the MOHCC´s daily situation reports. There was also an increase in the number of fatalities and there were anecdotal reports of public health sector facilities being overwhelmed. The actual reasons for the spike are still unknown, but this coincided with a general increase as was observed globally, and especially as mutant strains were discovered [6]. In particular, South Africa noted a mutant strain that reportedly had a higher effective reproduction number and more aggressive, with rapidly progressive clinical disease [6]. Unlike in the first wave, where the majority of cases were asymptomatic, there was an increase in the number of cases requiring hospitalisation and oxygen in the second wave, clearly depicting a change in behaviour of the disease.

The epidemiological trends of the second wave were not adequately explored, though the MOHCC produced daily and weekly situation reports. Understanding trends is critical for appropriate strategies to effectively control the current wave whilst avoiding further waves in the future. We therefore describe and explore the December 2020 to January 2021 trends, and discuss the implications for future public health direction.

## Methods

Secondary data were collected and collated from the daily situation reports produced by the MOHCC from 01^st^ December 2020 to 31^st^ January 2021. The reported variables were entered onto Microsoft Excel including the number of tests done daily, the new and cumulative cases and deaths, recoveries, source of cases and distribution of cases by province.

**Statistical analysis:** the data were then analysed in Stata 16.0 to depict trends. Frequency tables and graphs were produced in Stata, and variables that were not part of the daily situation reports were not included. All graphs were generated in Stata.

**Ethical considerations:** no ethical permissions were considered necessary for this secondary data analysis. There was no contact with participants and no individual participant data were collected, therefore there was no need for any individual informed consent seeking.

## Results

**Figure 1:**
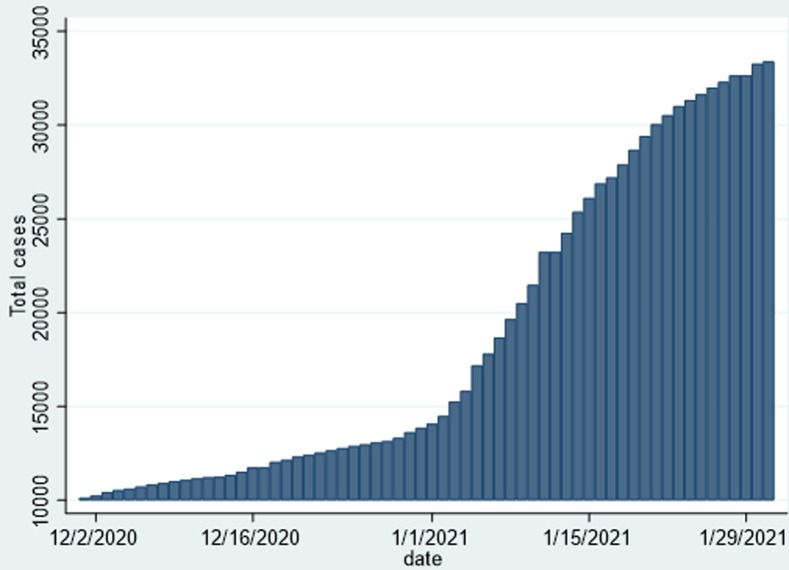
cumulative cases of COVID-19 in Zimbabwe from 01^st^ December 2020 to 31^st^ January 2021

**Figure 2:**
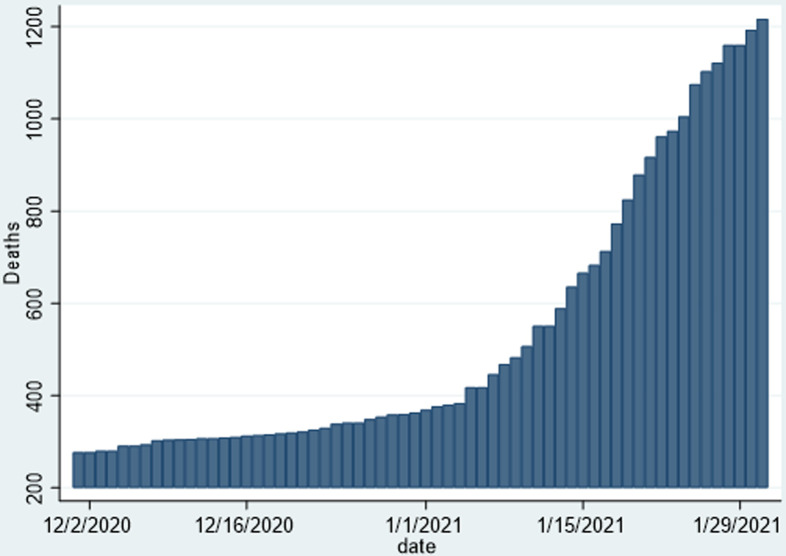
cumulative numbers of deaths attributable to COVID-19 in Zimbabwe from 01^st^ December 2020 to 31^st^ January 2021

**Figure 3:**
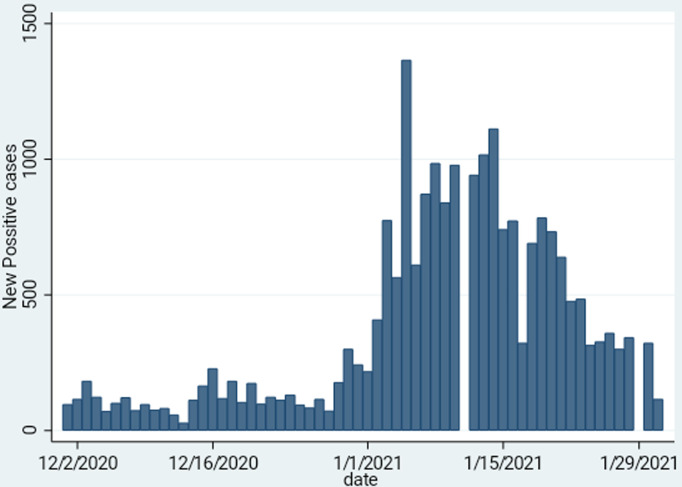
trends of the daily incident COVID-19 cases in Zimbabwe from 01^st^ December 2020 to 31^st^ January 2021

As shown in Table 1, there was a sharp increase in the cumulative number of confirmed cases, from an absolute of 10,034 cases on 30^th^ November 2020, to 33,388 on 31^st^ January 2021, representing a 333% increase over a two-month period. There was a respective increase in the other parameters such as the recovered and active cases. There was a significant increase win the number of deaths from an absolute number of 277 to 1217, representing a 439% increase in the absolute number of deaths over a two-month period, thus the majority of deaths attributable to COVID-19 in Zimbabwe occurred over this period. However, the national case fatality rate (CFR) only changed by 0.8% from 2.8% to 3.6% and remained comparable to that in other countries. The December 2020 to January 2021 epicurve in Figure 1 shows the marked sharp rise in the cumulative number of COVID-19 cases over this period, and Figure 2 shows a corresponding rise in the number of reported deaths. Figure 3 shows the trends in daily incident cases between 01^st^ December 2020 and 31^st^ January 2020 and shows that a peak was reached in early-January 2021, and then the incident cases started declining, around a period that coincided with two weeks into a newer stricter lockdown depicted as level four. The sharp spike noted coincided with the period of increased local and international mobility. However, Zimbabwe never experienced the pattern of exponential rise that characterised the pandemic in other countries.

**Table 1:** COVID-19 epidemiological indicators in Zimbabwe as of 30^th^ November 2020 and 31^st^ January 2021

Indicator	Number of cumulative cases as of		% Change
	30 November 2020	31 January 2021	
Cumulative Positive COVID-19 cases	10 034	33 388	333
Recoveries	8 489	26 044	307
Active cases	1 268	6 127	483
Deaths	277	1 217	439
RDT Antigen Tests	-	32 796	-
Total PCR Tests	169 183	301 198	178
National Case Fatality Ratio (CFR)	2.8%	3.6%	0.8

**Figure 4:**
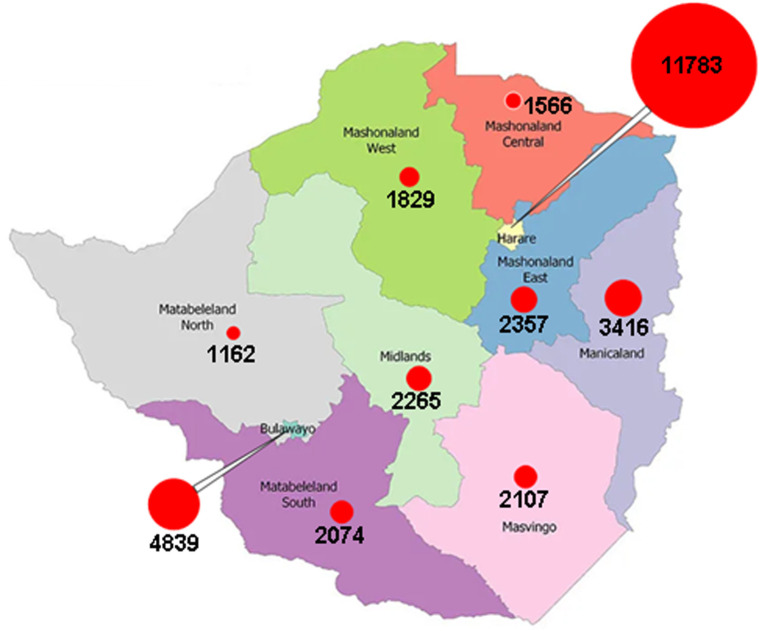
the spatial distribution of COVID-19 cases per province in Zimbabwe since March 2020

**Figure 5:**
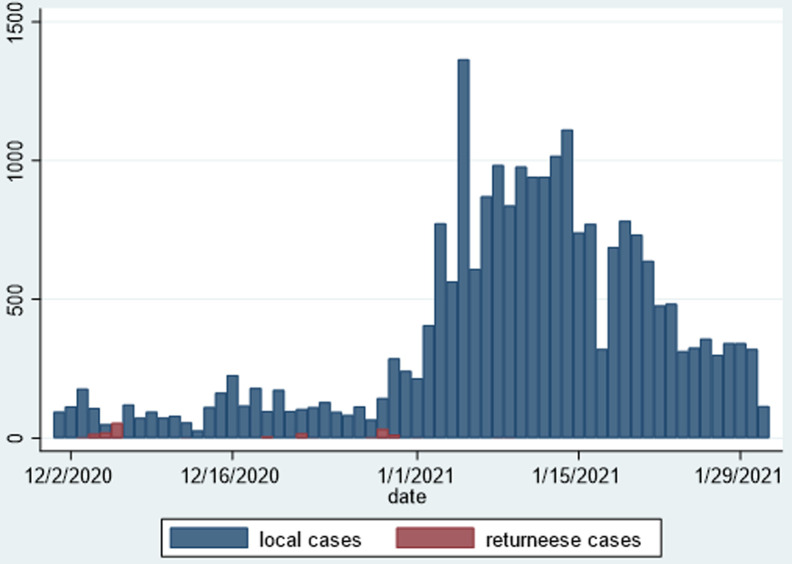
comparison of origin of people who tested positive for COVID-19 in Zimbabwe between 01^st^ December 2020 and 31^st^ January 2021

**Figure 6:**
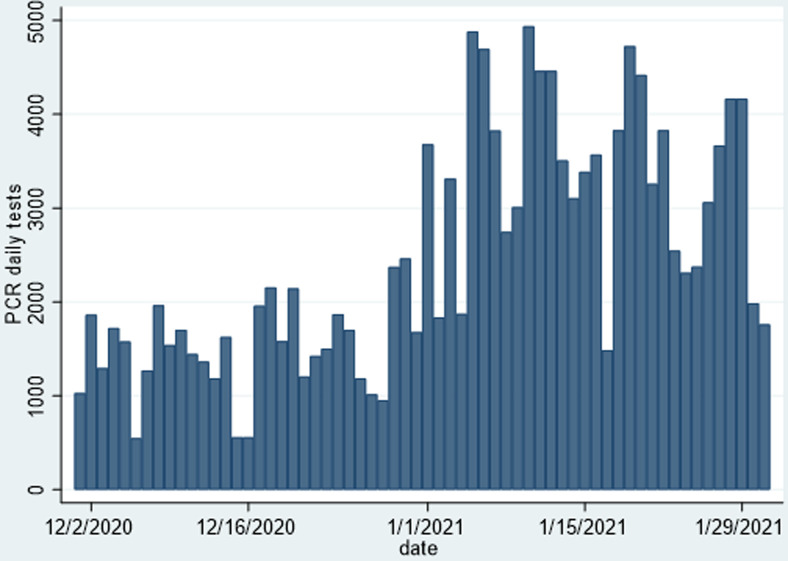
COVID-19 PCR tests conducted in Zimbabwe from 01^st^ December 2020 to 31^st^ January 2021

**Figure 7:**
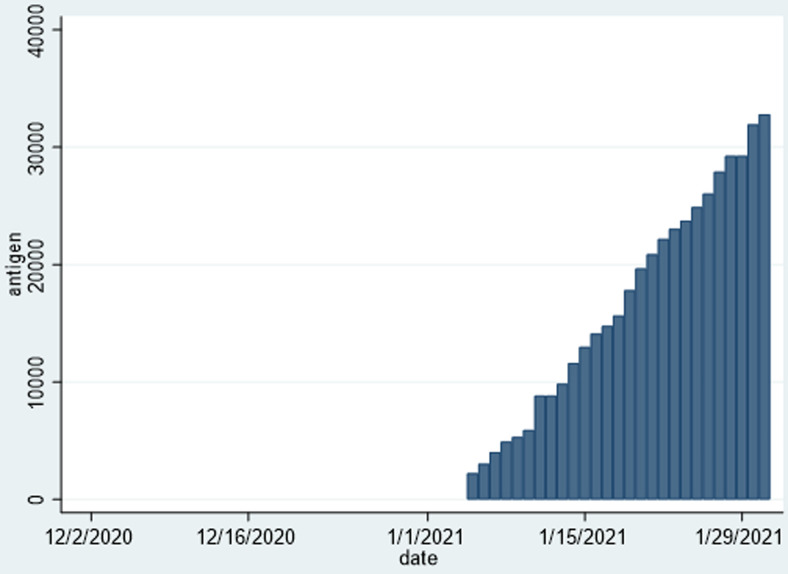
RDT antigen tests conducted in Zimbabwe from 01^st^ December 2020 to 31^st^ January 2021

Table 2 shows the distribution of COVID-19 cases per province from when the first case was reported in Zimbabwe in March 2020 to 31^st^ January 2021. Harare, Bulawayo and Manicaland were reporting the highest numbers of incident cases and fatalities daily. Harare Province accounted for 35.3% of the total number of cases, and 37% of the number of deaths. Combined, these provinces accounted for 60% of the total case burden, and the other seven provinces only contributed 40%. There was a respective representation in the reported number of case fatalities, necessitating differentiated interventions for COVID-19 control in these three provinces. Figure 4 is designed to show the spatial distribution of these cases, and the density of cases per province, and shows that Harare Province, has the highest density of cases per unit population. Figure 5 shows that by the greatest majority of cases over the period from 01^st^ December 2020 to 31^st^ January 2021 were confirmed among people with no history of travel outside Zimbabwe. Rapid diagnostic test (RDT) antigen tests were introduced formally into the testing strategy, and started to be included in the daily sitreps from early January 2021. There was an increase in the number of combined daily tests from less than 1000 per day at the beginning of December 2020 to a maximum of close to 5000 tests per day by mid-January 2020. Figure 6 and Figure 7 show the distribution of polymerase chain reaction (PCR) and RDT antigen tests over the concerned period.

**Table 2:** distribution of COVID-19 cases in Zimbabwe per province from 20^th^ March 2020 to 31^st^ January 2021

Province	Cum. Cases	Recovered cases	Active cases	Deaths
Bulawayo	4839	4215	438	186
Harare	11783	9165	2158	450
Manicaland	3416	2506	746	164
Mashonaland central	1566	1214	287	65
Mashonaland East	2357	1700	569	88
Mashonaland West	1829	992	713	114
Midlands	2265	1825	393	48
Masvingo	2107	1875	170	62
Matabeleland North	1162	609	540	13
Matabeleland South	2074	1943	114	17
Total	33 388	26 044	6 127	1217

## Discussion

We have presented the trends of COVID-19 in Zimbabwe over a two-month period between December 2020 and January 2021. This period represented a drastic shift in the pattern of the epidemic in the country, and is markedly different from what we witnessed in the first wave [4]. It has been widely speculated that increasing numbers of returning residents were responsible for the sharp rise in the daily number of COVID-19 incident cases over the festive period in Zimbabwe. However, our analysis showed that cases started rising gradually from early December 2020, reaching a peak in mid-January 2021. In-country mobility including city-to-city and urban-rural travel increased, and there were reports of large unsanctioned gatherings in the country. When a tighter lockdown was introduced in January 2021, the incident cases started to fall about two weeks later. Whilst, we cannot totally attribute the current level of control to the lockdown, this fall may be evidence that lockdowns do contribute to pandemic control. However, an in-depth analysis of why there was a sudden decline in the incident cases is warranted to inform future public health interventions for control.

The second wave of the COVID-19 pandemic, similar to the first wave [5], differentially affected the provinces. Harare, Bulawayo and Manicaland provinces reported the highest numbers of new cases and deaths [4, 5]. Increased human mobility and higher levels of socioeconomic activities, coupled with higher population density may be explanatory variables for this trend. However, it may be argued that increased access to testing may account for the higher numbers of cases reported in the bigger cities. Population wide seroprevalence assays may be required to draw worthwhile comparisons of these discrepancies. However, based on our study findings, and results from our previous analysis [4], differentiated strategies for control are required. The bigger cities with higher population density and more dynamic young people need tighter control. A previous study suggested that a younger, mobile and active population in Africa might be responsible for more asymptomatic COVID-19 transmissions [7].

There was a rapid increase in the numbers of reported daily cases, with the country reaching its first ever highest spike of over 1000 cases in one day in January 2021. The majority of the cases were attributable to community transmission, meaning these were people with no history of recent travel outside the country. Despite a marked increase in the absolute number of cases, the CFR was 3.6%, which is similar to the 3.6% CFR reported in a previous analysis for the period 20^th^ March 2020 to 30^th^ June 2020 [4]. This CFR is also comparable to what has been reported in other settings [8]. Ongoing debates regarding the justification of lockdowns for a disease with this low CFR in countries where more people die annually from other diseases such as malaria, tuberculosis and other infections remain unresolved [9]. The number of recovered cases was in excess of 89% and corroborates the low case CFR of COVID-19.

The MOHCC includes the numbers of admitted people with asymptomatic, mild, moderate, severe and critical disease in the daily sitreps, and these remain low. Asymptomatic people and those with mild-to-moderate diseases are sometimes admitted in institutions when their home conditions are assessed as unsuitable for isolation. Testing, treating and isolating confirmed cases is a critical component of breaking chains of SARS-CoV-2 transmissions. There were widespread social media reports of overwhelmed health facilities and people turning to home treatments and remedies for COVID-19 as they failed to access medical treatment in public and private health facilities. These reports were never verified; however, they should not be ignored. They potentially point to a strained public healthcare system with markedly diminished capacity to deal with increased disease burden. Health system preparedness to deal with emerging emergencies is critical as more disasters may arise in the future. African public health systems had been found wanting even in the first pandemic, but were expected to have adequately prepared for further waves [10]. To avoid straining fragile public health systems with increased disease burden, primary prevention must remain at the core of epidemic control. We must ensure continued adherence to prescribed infection prevention and control guidelines. These include, but are not limited to mandatory wearing of facemasks, physical distancing, frequent washing of hands with clean water and liquid soap or use of sanitizers, and practising good cough etiquette.

Zimbabwe has joined the rest of the world in implementing vaccination. Vaccination is the best public health approach to deal with the spread, morbidity and mortality associated with infectious diseases, and is considered as one of public health´s success stories. A phased approach to the vaccination programme starting with healthcare workers and others considered as frontline workers has been started, and will gradually be cascaded to the rest of the population. We hope that a sufficient proportion of the population will be vaccinated to attain herd immunity, reduce the morbidity and mortality associated with the disease, and reduce the strain on scarce healthcare commodities. Therefore, the Zimbabwean public health officials have a mammoth task of dealing with vaccination hesitancy to speed up acceptability and uptake of SARS-CoV-2 vaccines. Unfortunately, reports indicate that South African 501Y.V2 is now 60% predominant in Zimbabwe, and the efficacy of the current vaccines against mutant strains is largely unknown for now [6].

The trends show a current downward trajectory of confirmed cases. This may lead to relaxation of lockdown restrictions including increased mobility. Children have been out of school for a year now, which disadvantages them in terms of learning. Other economic sectors are also looking forward to opening as they have suffered from the ongoing lockdown. Lockdowns may be necessary for disease control; however, they are negatively impacting several socioeconomic aspects of people´s lives [9]. Guarding against complacency is thus critical at this point, and especially as the economy reopens. The Risk Communication and Community Engagement (RCCE) Pillar co-Chaired by the MOHCC and Ministry of Information has an arduous task of reinforcing the need for people to continue observing prevention protocols in all spheres. The country must avoid as much as possible further waves of COVID-19 and lockdowns which are detrimental to other aspects of life [9].

## Conclusion

The period from 01^st^ December 2020 to 31^st^ January 2021 was a defining period for the COVID-19 outbreak in Zimbabwe, which saw a steep rise in both the confirmed number of cases and fatalities attributable to the disease. Increased human movement over the festive holiday led to increased transmissions rates. Policymakers must aim to identify critical periods when there is likely to be increased transmission, and put in place adequate public health interventions to limit potential explosions and further waves of COVID-19. Differentiated approaches to control the spread of COVID-19 in the hardest hit provinces are required to tighten control, whilst allowing a reasonable level of socioeconomic activities in the other provinces. It is also hoped that vaccination will add another desired layer of protection and help to put the pandemic under control.

### What is known about this topic

- Increased, uncontrolled human movement leads to increased transmission of SARS-CoV-2;
- The case fatality of COVID-19 is generally low;
- Adhering to prescribed infection prevention and control protocols such as facemasks, frequent hand hygiene and physical distancing significantly reduces the burden of COVID-19.

### What this study adds

- Increased local movement of people was responsible for the widespread community transmission of COVID-19 which occurred in Zimbabwe between 01^st^ December 2020 and 31^st^ January 2021;
- Despite an increase in the absolute numbers of deaths, the case fatality rate of COVID-19 remains low at 3.6% in Zimbabwe;
- Differentiated strategies of control are needed to effectively control the spread in the high-risk provinces of the country, whilst allowing reasonable levels of activities to occur in low-burden provinces.
